# Trauma of bone and soft tissues in South American mummies—New cases provide further insight into violence and lethal outcome

**DOI:** 10.3389/fmed.2022.962793

**Published:** 2022-09-09

**Authors:** Anna-Maria Begerock, Robert Loynes, Oliver K. Peschel, John Verano, Raffaella Bianucci, Isabel Martinez Armijo, Mercedes González, Andreas G. Nerlich

**Affiliations:** ^1^Instituto de Estudios Científicos en Momias, Madrid, Spain; ^2^KNH Centre for Biomedical Egyptology, University Manchester, Manchester, United Kingdom; ^3^Institute of Forensic Medicine, Ludwig-Maximilians-University, Munich, Germany; ^4^Department of Anthropology, Tulane University, New Orleans, LA, United States; ^5^Department of Cultures and Societies, University of Palermo, Palermo, Italy; ^6^The Ronin Institute, Montclair, NJ, United States; ^7^Institute of Pathology, Academic Clinic Munich-Bogenhausen, Munich, Germany

**Keywords:** forensic investigation, fractures, skull trauma, stab wound, pseudopathology

## Abstract

There exist numerous reports on violence in South American populations which shed a particular light on life and living conditions in those historic communities. Most studies have been performed on collections of isolated skulls. Whole-body investigations especially on well-preserved mummified human remains are rare. In the present study we investigated three South American mummies predating the Colonial Spanish period. The “Marburg” man lived between 996 and 1147 CE and was buried in typical burial bundle. The analysis of the textiles, ceramics and fishing tools associated with his naturally mummified body suggests that he most likely originated from the Arica region in Northern Chile and was possibly part of a fishing community. The “Delémont” natural mummies belong to an adult male and an adult female, respectively. The mummies, the textiles and grave goods were investigated. The ceramics suggest a provenance from the Arequipa region, supposing that all the artifacts were originally associated with the two mummies. The Delémont male mummy is ^14^C dated between 902 and 994 CE and the “Delémont” female mummy ^14^C dated between 1224 and 1282 CE. All mummies underwent Multidetector Computed Tomography which showed evidence of trauma, some of which were interpreted as evidence of interpersonal violence. An interdisciplinary approach was applied with the particular intention to identify trauma sequels and to evaluate their paleo-forensic potential. Evidence of violence was identified in the two male individuals. Our study provides evidence that the interdisciplinary investigation of well-preserved human remains may detect much more frequent traces of intentional trauma than previously thought. Particularly, trauma against the body may not be identified in studies on skulls alone, and trauma residues of internal organs/soft tissues will only be seen in mummies. We therefore add further evidence of two cases of (lethal) trauma in pre-colonial South-American male individuals.

## Introduction

In pre-Columbian South-American history, there is significant evidence for trauma and intentional violence in various geographical areas and different levels of social organization, although no data indicate a particularly high epidemiological level of trauma. This holds true for cases in warfare, but also arises in cases of interpersonal conflicts and overt murder (1–8). Beyond most studies on a single-case basis which do not allow more generalized estimations, a recent review of pre-Columbian Chilean samples found trauma rates of between 5 and 35.6% of individuals (8). Unfortunately, most studies have been performed only on human skulls (from collections that contained only cranial material) and few studies exist that include whole body human remains (mummies). This renders the aforementioned data difficult to compare and interpret. Likewise, in skeletal material only trauma affecting bone can be identified, while in mummified human remains perimortem trauma with soft tissue involvement may be identified as well (6, 9). This sheds additional light on the occurrence and frequency of intentional injuries as well as the type of lesions and their pathomechanisms. In contrast, any possible traumatic lesion must be evaluated with care in order to rule out post-mortem breaks and pseudopathologic conditions that may mimic trauma, in order to avoid misidentifications and misinterpretations.

This paper describes three hitherto unreported natural mummies held by two European collections: one mummy is kept at Marburg University, Germany, while the other two bodies are currently housed in the Delémont Museum, Switzerland. The interdisciplinary investigation of these three natural mummies shows evidence for pre- and perimortem trauma, partly accompanied by post-mortem breaks. Postmortem damages to the bodies are mainly due to nineteenth century brutal excavations of South American pre-Columbian mummies.

Taking this ensemble of individuals, we provide an insight into the usefulness of interdisciplinary analysis of mummified human remains and, particularly, the potential of transdisciplinary interpretation of biological findings.

## Materials and methods

### Basic description of the mummies, their origin and provenance

#### The South American “Marburg mummy”

**FIGURE 1:**
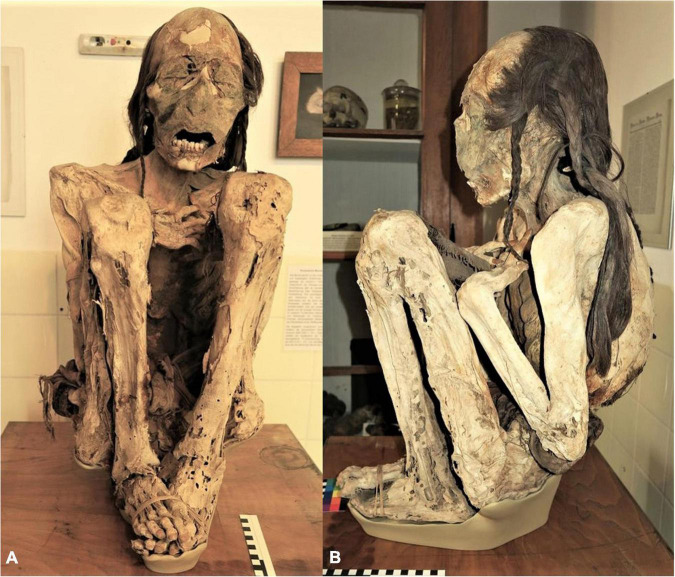
The “Marburg mummy”—Macroscopic views of the whole mummy. **(A)** Frontal and **(B)** lateral view.

Amongst the specimens of the “Museum Anatomicum” of the Philipps Universität Marburg, Germany, Medizinische Fakultät, a pre-Columbian mummy (Inv. No. 1315.1.2.) is exhibited in the permanent exposition. The individual had been buried in a seated and flexed position (Figure 1) which includes placement inside a burial bundle. This was a common burial pattern in pre-Columbian Peru (10). The associated burial goods originally may have been placed inside the bundle or around it, but during excavation and further handling this “composite bundle” was disrupted. Nevertheless, all available artifacts have been re-united with the mummy and are now presented in the same showcase. Despite these “reconstructions,” the mummy is naked, bereft of the usual burial bundle and all of its clothes, which is also typical of display practices in some museums.

Since the inventory information of this mummy is very detailed it is fair to assume that mummy and burial goods belong to the original burial setting –something that is rare in other mummies from South American collections. Furthermore, the available data indicate the year of acquisition and its excavation site. The accession record reads as follows “*The Anatomical Institute received as a gift from the physiologist Rudolph Eduard Külz, who worked for the University of Marburg, a well preserved female mummy from Arica, along with a number of items that have been found with the mummy: a good quantity of larger and smaller clay pots, various other household items, yarn, fabric, spindles, several pelican eggs as well as some peculiar objects that are called ‘ojos de gentile*s’ [‘gentle eyes’] …” (Entry in the Chronicles of the Marburg University, financial year 1892/93, translation from German by the authors).

As recent research has shown, the mummy seems not to have been scientifically investigated since the date of its arrival in the collection. However, as part of an ongoing mummy research project by the Instituto de Estudios Científicos en Momias, Madrid, Spain (IECIM), the mummy has now been extensively examined, following a research agreement with the Museum Anatomicum. The team was able to determine the sex of the individual as male, contrary to the ascription in the Chronicles of Marburg University, and to study its accompanying burial goods. Based on this recent examination, the individual belonged to the Arica culture, flourishing in what is today Northern Chile, between 1000 and 1470 CE. A radiocarbon determination confirms this [555 ± 33 BP (Mams 15,174), calibrated with Oxcal 4.3.2 and the ShCal dataset, 2 sigma, to date between 996 and 1147 CE].

A detailed analysis of the textiles and ceramics associated with the mummy provides further evidence that this mummy originates from the Arica region in Northern Chile and most probably had been legally sold in 1892 together with the aforementioned burial artifacts. The individual once lived in a fishing community, as most of the artifacts are fishing tools (10, 11). There is no evidence that the individual was a warrior nor are there indications for distinct ritual functions.

#### The South American Delémont mummies

**FIGURE 2:**
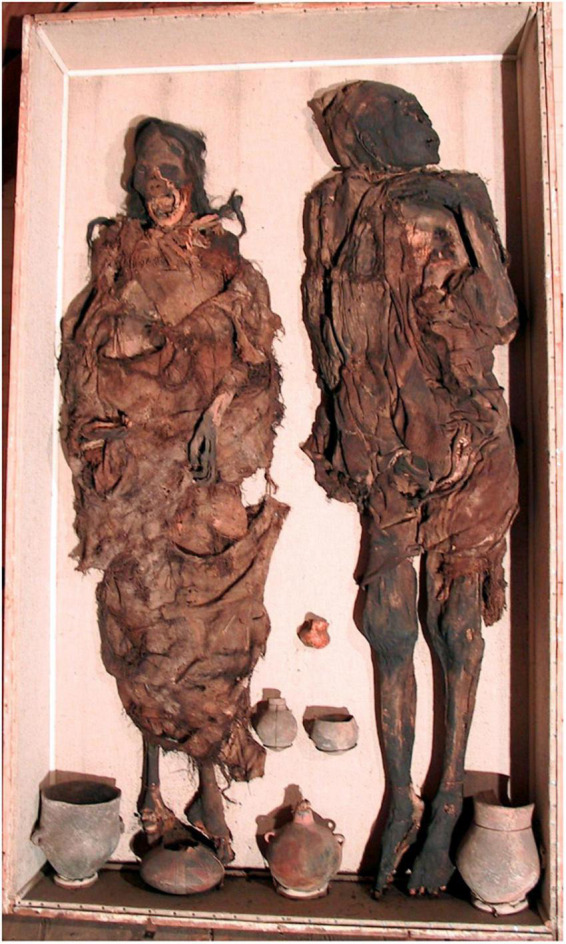
The “Delémont man” **(right)** and the “Delémont woman” **(left)**—“overview of the two mummies in their repository case.”

The Musée jurassien d’art et d’histoire (MJAH) in Delémont, Switzerland, is today’s home of two South American mummies: one male (Figure 2 right; Inv. No. MJ.1996.11.2) and one female (Figure 2 left; Inv. No. MJ.1996.11.1). Both individuals are not presented in a permanent exhibition, but kept in the museum’s repository. There exists no written reference to the date of acquisition, but both must have come to the collection before 1866, since in that year the Collège de Delémont was built, including a museum with a large collection of various “curiosities” (12). There exists no further information about the detailed provenance or the collector. Several burial goods originating from the Arequipa region were possibly acquired with the two bodies; all these goods are part of the museum’s collection and are associated to the bodies. Whether they belonged to the dead or not, has not been so far clarified.

**FIGURE 3:**
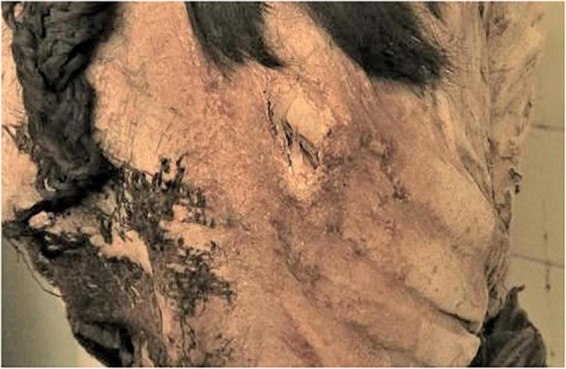
Detailed view of the dorsal thorax of the “Marburg mummy” showing the defect. The margins of this defect are slightly rounded.

Both mummies are quite peculiar since they were buried in supine position (see Figures 2, 3), a seemingly atypical position for highland South American mummies. In most cases, from around 1000 CE onward, pre-Columbian individuals were buried seated. However, it shall be underlined that supine burials were common in cultures of the Western coast of South America prior to AD 1000 [Middle Andean Phase 1B, see (13)]. Radiocarbon dating of the male mummy provides a date of 1076 ± 16 BP, or following calibration (oxcal 4.3.2 and the ShCal dataset, 2 sigma) a date between 902 and 994 CE, in accordance to this phase of burials in supine position. Interestingly, the radiocarbon date for the female mummy differs, producing a date of 798 ± 16 BP, and a 2 sigma calibrated date of 1224–1282 CE, thus dating later than the male mummy and later than the period when burials in supine position were most frequent. Nevertheless, the female mummy is a typical South American mummy and the divergent radiocarbon dates may indicate that the male and the female mummy were not related to each other.

Both individuals are covered with textiles made from cotton and camelid hair. Due to their arrangement and intimate contact with the mummified skin, a careful inspection suggests that these textiles were placed on the cadavers shortly after death. However, it cannot be completely ruled out that later manipulations occurred, an issue that remains unresolved. Despite this remaining minor uncertainty, it is clear that the use of camelid hair for the ribbon for the hair of the woman, in its original position, further confirms its pre-Columbian origin, as does the Vizcacha hair for the woolen bonnet of the man. Both types of animals did not exist elsewhere beyond South America prior to the Spanish Conquest and later trade (10). The mummies were investigated macroscopically, as well as the textiles and grave goods. The ceramics suggest a provenance from the Arequipa region, if all the artifacts were originally associated with the two mummies (11).

### Paleoimaging

The three mummies underwent paleoimaging by whole body CT scanning in order to determine their state of preservation, sex, age, and any skeletal or soft tissue pathology and, eventually, to determine the cause of death. The determination of age, sex, and stature were performed using standard anthropological methods (14–16).

The “Marburg mummy” was investigated with the kind support of the Radiologische Klinik, Universität Marburg (Hesse, Germany) using a Siemens Somatom Definition machine. The investigation was performed under routine conditions with a slice thickness of 0.75 mms and 80 kv in standard algorithm. The resulting 1,825 slices were further investigated for multiplanar and 3-D reconstructions as usually performed (6).

In parallel, the Delémont mummies both were analyzed at the Institut für Klinische Radiologie und Nuklearmedizin—Universität Mannheim, Mannheim (Germany), again using a Siemens Definition machine with a slice thickness of 1.5 mms (male mummy) and 0.6 mm (female mummy), and 80 kv, standard algorithm. Again, multiplanar and 3-D reconstructions were performed as indicated before (male mummy in 1,777 slices, female mummy in 1,432 slices).

Age was estimated through the analysis of different parameters (17), these were obtained from the CT scans and included evaluation of the closure of the epiphyses, changes of the pubic symphysis and auricular surfaces and modification of the trabeculae of long bones (14–16). Particular attention was paid to the closure of cranial sutures since these have recently been re-evaluated for post-mortem age determination in groups of > 40 years, 40–60 years and < 60 years (18). Stature was estimated measuring the maximum lengths of femurs on the CT images and applying Ruff et al. (16) Equation. The presence of degenerative joint and spine alterations was also evaluated and taken into the account of age estimation, such as the wear and tear of the dental enamel.

Further information obtained by paleoimaging comprised the activity patterns of joints and spine, and especially pathological features of the complete bodies, including trauma and their sequels, post- inflammatory changes, metabolic diseases, calcifications, especially those of the vasculature.

### Forensic evaluation of the cases

In a subsequent step, both macroscopic and CT-scanning observations of trauma were re-evaluated under the aspects of the underlying patho-mechanisms with particular reference to potential forensic implications. These evaluations were done as a case study conference between the various subspecialities involved (anthropology, radiology, forensics, pathology).

The most relevant criteria were: discontinuity of bones (fractures/breaks, either pre- or postmortal); eventual bone reaction (e.g., evidence for healing/reactive periostitis); soft tissue defects (especially defects of the skin surface); internal organ defects, including changes in radio-opacity of internal structures, defects and malposition of organs and structures.

## Results

### The “Marburg mummy”

#### External (anthropological) examination

A concise macroscopic investigation (see Figure 1) revealed in this case clearly a very well-preserved male individual as evidenced by male external genitalia (which differs from the initial indications by the acquirer and the corresponding remarks in the chronicle “female mummy from Arica”). The body was placed in a typical seated position. At present, it is covered by an almost intact skin surface with minor defects at the extremities. The arms are crossed over the abdomen. Textile impressions on the skin indicate that the face had been covered post mortem by a tunic, that also covered the rest of the body. There are further pieces of fabric attached to the surface of the mummy, especially at hip height at the mummy’s back. A separate textile covers the genitalia. The forehead is bald; the occiput is covered by long, full dark hair woven in several long plaits. Both eye lids are closed. The mouth is open; a series of well-preserved, though misaligned teeth 31, 32, 41, 42, 33, 43, 34, and 44 are observable in the lower jaw (19). All teeth do show minor abrasion, as is typical for pre-Columbian populations with maize as their main staple food (10). Finger and toe nails are present, although the toes have partially lost their soft tissue.

On detailed examination, at the right dorsal thorax, a slightly oblique skin defect of approx. 3.5 cm length with rounded margins which runs along the 8th rib can be identified (Figure 3). No further defects, nor evidence for a post-mortem opening or any other manipulation are present.

#### General findings by paleoimaging

In addition to the visual identification, CT scan images further confirm that the individual was a male by skeletal masculine features such as prominent brow ridges and muscle insertions at the skull, a narrow sub-pubic angle and narrow sciatic notches. According to the afore indicated skeletal criteria, the age at death is assumed to range between 20 and 25 years which is confirmed by the lack of fusion of the sternal end of the clavicle (14–16). The morphology of the dentition with rounded crowns and a partially erupted upper right third molar (tooth 28) further supports the notion that this individual was a young adult. The upper left 2nd premolar (tooth 25) was lost postmortem (19). The measurement of the stature indicates that the man was ca. 172 cm tall (16).

**FIGURE 4:**
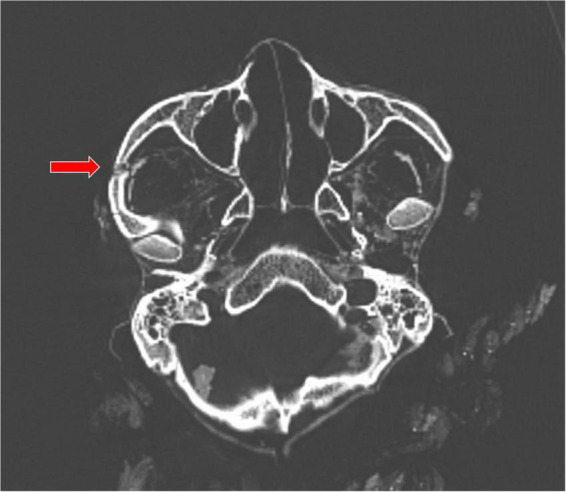
The right zygomatic fracture (arrow).

Whilst there is a slight depression in the skull across the region of the coronal suture, there is no evidence for a dolichocephalic deformation which is frequently seen in South American mummies (20). Within the skull focal residues of preserved brain tissue and the meningies are seen. The desiccated eye globes are preserved *in situ*. Two fractures of the right zygoma, one of which is displaced, can be appreciated (Figure 4). There is, also, a dislocation of the left temporo-mandibular joint with anterior translation of the left mandibular condyle. No evidence of healing in the zygoma can be seen. There is no further ligamentous dissociation. The cervical spine and soft tissues of the neck are normal.

**FIGURE 5:**
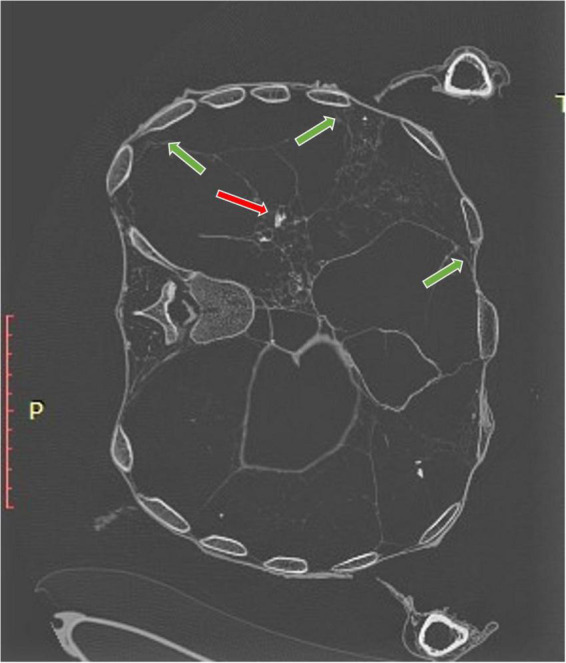
CT scan of the thorax: the lung parenchyma is focally condensed and shows multiple small calcifications (red arrow). Furthermore, both lungs appear as widely attached to the thorax wall (green arrows) suggesting post-inflammatory pleuritic adhesions.

As to the thoracic region, the chest is barrel-shaped as a result of the final posture of the body. Except for the right upper field, both lungs are expanded with extensive adhesions to both chest walls and the diaphragm. The parenchyma itself is irregularly condensed and shows numerous variably large calcifications between few millimeters up to 1 cm (Figure 5). These are observed in both lungs in central and focal peripheral regions with particular reference to the right lower and middle lobe and the left lower lobe. In accord with the morphology and distribution these are almost pathognomonic for the residues of an extensive, old-healed inflammation (most presumably of tuberculous origin, therefore so-called post-specific residues). The pericardium is well retained, however, there are no residues of the heart muscle which is not unusual in naturally mummified individuals (21) and which may be the result of ongoing decomposition processes not relevant in more “recent” postmortem autolysis studies (22). The bronchial tree is well identified, as is the aorta. The latter does not show any evidence for arteriosclerosis, in particular no calcifications. The diaphragm is intact.

**FIGURE 6:**
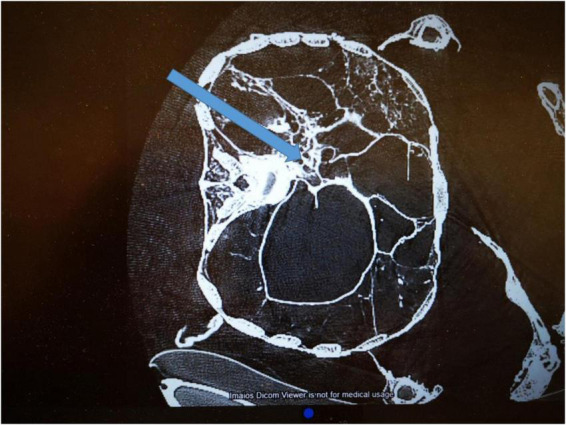
CT cross-section of the thorax at the level of the dorsal defect. The arrow indicates the presumed direction of an obvious stabbing wound reaching to the aorta and being surrounded by some major radiodense material.

At the posterior thorax wall the macroscopic defect is identified with slightly inward turned margins as described. The defect affects the right posterior wall of the thorax (8th rib). On sagittal sections (Figure 6), this defect extends into the thorax affecting the right lower lobe at a site of focal right lung collapse and ending in an area of high radio dense material highly suggestive of (desiccated) blood residues just adjacent to the descending aorta. The aorta seems incorporated into the bleeding masses; this is consistent with a hemo-pneumothorax secondary to a stab wound in the back with extensive bleeding residues due to a laceration of the descending aorta. There is no evidence of any foreign material in this location.

The outlines of intra-abdominal structures are visible, but there is little evidence of solid organs. There are no abnormalities in the bony pelvis, hips or the spine except for a post mortem lumbar postural kyphosis.

##### Evidence for further postmortal “preparations” of the mummy

In the CT-scans one finds two “pouches” fashioned from textile material lying just below the waist in the posterior region of the hips. These have been prepared in order to “stabilize” the squatting position of the mummy. They obviously have been added post mortem and have no relation to the mummy during lifetime or at the period of death.

#### Medico legal considerations

There is no doubt that the individual of the Marburg mummy was a victim of a severe and finally lethal interpersonal violence. This comprises two complexes of observations: First, the fracture of the right zygoma is consistent with a very circumscribed fresh post-traumatic fracture (without evidence for remodeling/healing) following a heavy pre-/perimortem blow to the right upper face. There is no evidence of trauma of the right mandibula, but a dislocation of the left temporo-mandibular joint which may have happened as a certain “contre-coup.” This dislocation was then fixed in its position during the drying process of the mummification so that a later post mortem dislocation can be excluded.

Secondly, there is circumstantial evidence for a stabbing to the posterior thorax using a slender cut-and-thrust-weapon that perforated not only the skin, but also the lung parenchyma and most likely even lacerated the descending aorta. This must have led to a massive and rapid extensive blood loss (via the stab wound track) with some residues still being localized in the right lung. The effects of the stabbing and the bleeding seem to have been further influenced by the pathologic pre-conditions of the lung parenchyma which shows extensive laminar adhesions of the two pleural membranes on both sides, focal calcifications and fibrosis. This strongly suggests an extensive old-healed post-inflammatory pleuritic adhesion, most presumably due to bilateral post-specific inflammatory infiltrations of the pleura, such as e.g., in tuberculous infection. Furthermore, within the right lower lobe some irregular deposits of dense material are seen which might be (dried) bleeding residues.

The dorsal thorax wall defect is consistent with a penetrating stab which entered the expanded right lower lung. Accordingly, a single-strike laceration of the aorta would have led to a rapid loss of blood pressure due to a massive immediate blood loss; as a consequence, the Marburg man lost consciousness within a few seconds and died without any type of defensive reaction.

In conclusion, the individual received several perimortem lesions with at least one strike against the head and a single deep thoracic stabbing which lacerated the aorta and was most presumably the ultimate cause of his death.

### The Delémont male mummy

#### Anthropological (external) examination

**FIGURE 7:**
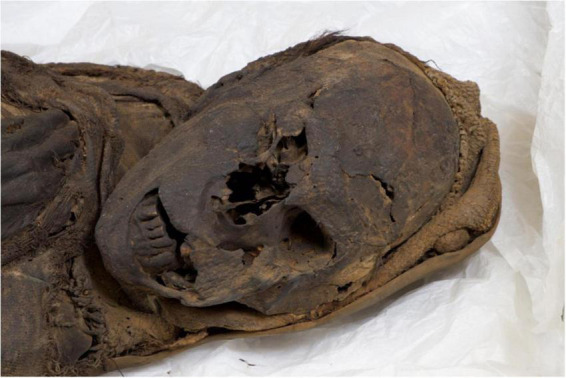
The Delémont male mummy: view of the face and aspects of the upper body half from the right side.

The adult man (inv. no. MJ.1996.11.2) was buried and spontaneously mummified in supine position (see Figure 2 right). Today his body is partly covered by fabric made of cotton and Camelid hair, the backside of the head is covered with a woolen bonnet, made with hair from Vizcacha, a mountain hare. The mummy itself is well preserved and shows only minor defects of the skin, e.g., at the toes and some minimal defects at the face (Figure 7). The face is turned to the left side, the right arm is stretched along the body, the left arm crossed over the chest. Male external genitalia are preserved. There are no signs for an opening of the body nor for other embalming manipulations. The skin is dark brown. The individual’s head is covered by the bonnet mentioned before. It is tied with a thin rope under the lower jaw. The eyes are retracted into the orbits; the mouth is slightly open showing some of the mandibular teeth. At the right orbit the skin surface seems defective with some destruction of skin and most probably the underlying bony part of the orbital cavity (Figure 7). Due to the covering and a modern crate underneath the mummy, no further details of the remaining parts of the body, such as the back, the thighs or the chest wall are visible.

#### General findings by paleoimaging

**FIGURE 8:**
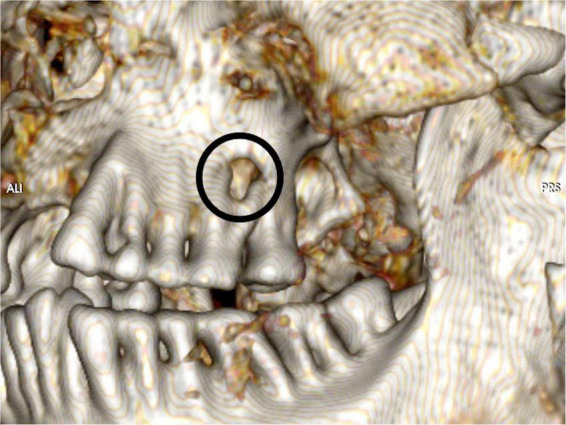
Detailed view of teeth at the left maxilla shows a dental apical defect very suggestive of an apical dental (root; tooth 26) abscess (circle).

The CT-scans images confirm the aforementioned macroscopic findings including male sex. In addition, there is skeletal evidence of male sex as the pelvis demonstrates a sub-pubic angle of 65° and narrow sciatic notches. The prominent bony markers at the skull further support male sex. With respect to the individual age, mature adult age can be assumed: all epiphyses are fused, cranial sutures are partly fused suggesting an age of ca. 40–60 years. Tooth loss was extensive [teeth 11, 12, 21, 22, 28, 46, 48 (19)]. Additionally, the individual shows focal apical dental disease with abscess (tooth 26) (Figure 8). The root of tooth 47 is present while the crown has been fractured and lost. The measurement of the stature indicates that the man was 169 cm tall (16).

The cranial shape has been altered artificially, resulting in an indentation running around the skull from the vertex to the external auditory meatus bilaterally resulting in a skull shape verging on the dolichocephalic (cranial index = 70). The skull itself is extensively damaged by both recent and historical injuries.

**FIGURE 9:**
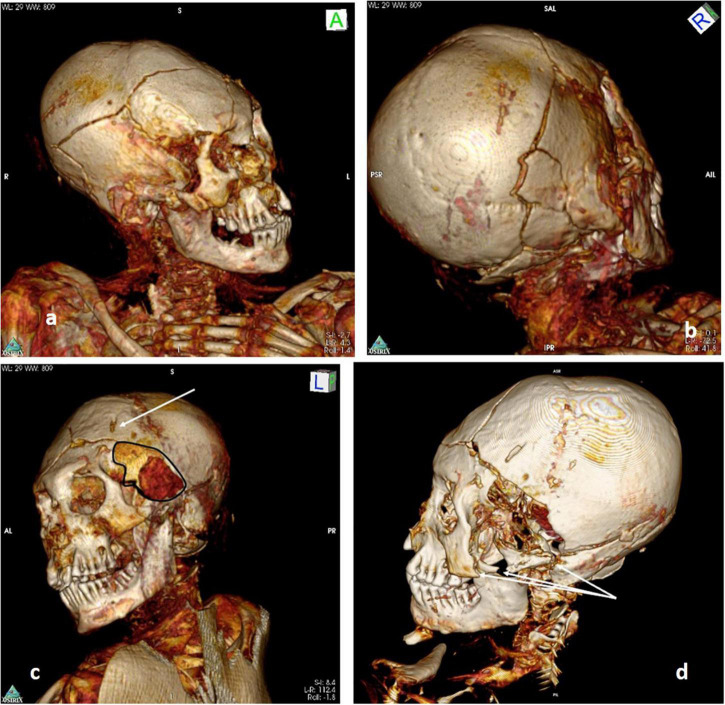
**(A–D)** Features of perimortem skull trauma to the left side of the skull with a large perforating lesion at the left temporal region (arrow) and a fracture running into the skull. **(D)** Shows the zygoma’s fracture of the left side (arrows).

**FIGURE 10:**
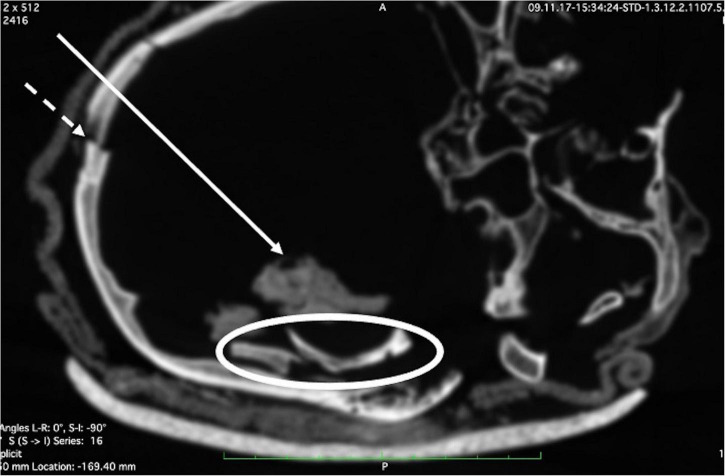
Some fragments from the external table of skull coming from the area of the lesion are seen within the skull cavity—lying close to dried brain tissue residues (full arrow). Note the fracture line (dotted arrow) at the dorsal cranium.

Recent injuries, close to the time of death, include multiple skull vault fractures (Figures 9A,B) and a large perforation in the left temporal region (Figure 9C)—measuring approximately 5.0 × 7.5 cm—accompanied by three fractures of the left zygoma (Figure 9D). Whilst the perforation may, arguably, have been enlarged by postmortem manipulations, the clue to its having been caused by a blow to the region is confirmed by the presence of bone fragments within the cranial cavity (Figure 10). There is some desiccated brain lying in the left occipito-parietal region of the cranial cavity.

**FIGURE 11:**
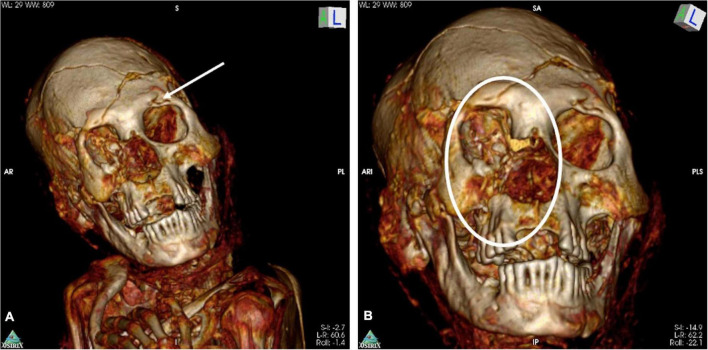
**(A,B)** Old healed defects to the left cranial orbital ridge (arrow) and major old healed lesion of the right orbit (circle).

Previous skull and facial injuries are also apparent, resulting in significant deformity. Figure 11A shows an old damaged area of the left superior orbital margin. Figure 11B demonstrates an old complex injury of the right side of the face including healed fractures of the orbit, maxilla and malar regions. The right side of the nose also appears to be missing. The deformity of the right maxilla and maxillary dentition results in malalignment of the teeth. Also, there is clear asymmetry of the orbits.

**FIGURE 12:**
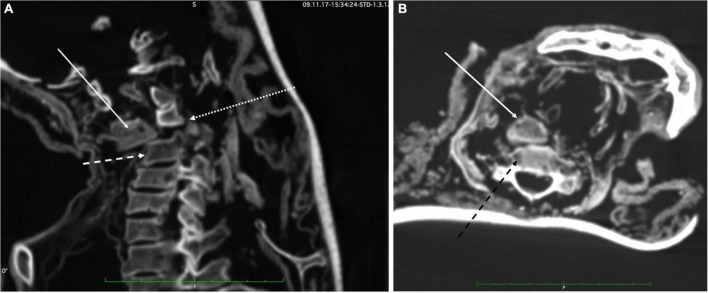
**(A)** Sagittal CT-scans through the cervical spine: There is a significant dislocation (small dotted arrow) between C3 (arrow) and C4 (dotted arrow) that may have resulted from a massive forward flexion. **(B)** The axial scan confirms the dislocation along with the surrounding intact soft tissue (white arrow = body C3; dark doted arrow = body C4).

In the cervical spine, degenerative change is evident at the C4/5 and C5/6 levels. However, the main finding in this region is a significant dislocation of the vertebrae at the C3/4 level. The cause of this dislocation appears not to be an artificial lesion, since the dried soft tissue around the lesion is well preserved which strongly argues against an artifact (e.g., during excavation or subsequent transportation). It is therefore fair to assume that it is the result of an intravital/perimortal trauma with a mild rotation plus violent flexion force (Figure 12) (for further interpretation see medico-legal discussion). Although there is evidence of a cord around the neck, this is neither continuous nor tight—as one might expect in a case of hanging. Additionally, there is no bone fracture, neither a typical hangman’s fracture. The perivertebral soft tissue does not show alterations nor major bleeding residues.

In the thoracic region, the right clavicle shows a mark on the shaft probably representing an incised injury, the bone being partially divided without, however, any tissue resection. A number of bony abnormalities including dislocation of the right 4th–10th costo-vertebral joints and fractures/breaks of the right 3rd–8th ribs laterally and the right 3rd posteriorly, can be identified. There are, also, fractures/breaks of the left 3rd–8th ribs laterally. The rib fractures could well be the result of an antero-posterior crushing injury. However, dislocation of the costo-vertebral joints is almost unheard of in living humans, and are most probably the result of taphonomy (postmortem dislocation and breaking events). Additionally, the fractures do not follow the typical lateral lines presenting a further argument for a postmortem effect. Remnants of the heart, mediastinum, lungs (typically retracted to the hilum) and a focal calcification of the aorta can be appreciated. The abdominal cavity contains the residues of several desiccated organs and soft tissues of the limbs are unremarkable. A slight calcification of the right *Arteria iliaca communis* is present.

The bony pelvis is intact and hip joints normal; the spine is normal apart from the abnormalities noted in the cervical spine.

#### Medico-legal evaluation

The Delémont male mummy shows several lesions suggestive of traumatic origin:

(a) A major skull defect of the left temporal region and adjacent skull fractures running from the defect into the skull bone. Since there is no bone tissue reaction at all, this lesion most likely occurred shortly before or at the time of death. Possibly, the perforating lesion was lethal.

(b) Three fractures of the left zygomatic arch with dislocation of a major bone fragment, in shape and lack of reaction very similar to (a); in consequence, the zygoma fractures possibly occurred before death.

(c) complex fractures of the contralateral right side of the skull with healed fractures of the orbit, maxilla and the malar region with some dislocation of the fracture lines and, therefore, deformation of the right midface; the right side of the nose is missing, possibly as a result of secondary loss of material at the old-healed fracture zone; this complex of lesions occurred significantly before death—an old-healed vital fracture complex with healing in a slightly displaced position.

(d) there is an impression of the left superior orbital margin which appears to be healed; this has no connection to the fresh fracture zone on the left side nor to the old-healed fracture residues of the right side. It may be an additional healed post-traumatic lesion and represents a second blow to the skull which may have occurred together with the right-sided attack (as described in c).

(e) There is a dislocation of C3/4 with “massive obliteration” of the spinal canal. There is no concomitant osseous lesion and, therefore, a massive direct trauma cannot be proven; however, the significant dislocation of the two vertebral bodies may have most presumably occurred during a flexion/rotation trauma, particularly in a relaxed/unconscious individual. This is atypical for hanging (also there is no hangman’s fracture). Accordingly, this lesion occurred most likely in a forward moving, but fixed individual, so that we may speculate that this lesion occurred perimortem in a stunned individual (e.g., by the blow to the skull).

(f) there are numerous serial fractures of ribs on both sides without signs of healing, a part of which shows a dislocation of the costo-vertebral joints; the almost symmetrical arrangement at the lateral side of each rib strongly suggests a post-mortem compression of the thorax.

(g) Lastly, there is a discontinuity of the right clavicle in its central portion with a gap between the two bone fragments which most likely occurred after death since there is no tissue reaction.

To conclude, based on the presence of vital reactions, we propose **recurrent trauma:** the healed right-sides skull fractures and the supraorbital impression fracture occurred considerably before death; conversely, the perforating left temporal skull trauma (and the zygomatic fractures) occurred perimortem. Also, the dislocation of C3/4 occurred perimortem and may be considered the cause of death. Both the bilateral rib fractures and the clavicular defect are more likely taphonomy related.

### The Delémont female mummy

#### Anthropological examination

This adult female individual (inv. no. MJ.1996.11.1) was also buried in a supine position (see Figure 2 left) as was the Delémont male mummy. The body is now almost completely covered by a roughly woven fabric. The arms are stretched along the body; the skull shows some defects at the mouth which is widely opened and there are some defects at both feet. The rest of the body is still tightly covered by the textile, and is therefore not accessible for further decisive macroscopic evaluation. The individual’s long dark hair was arranged in two braided plaits.

Since external inspection is limited, the genitalia are not visible, so that the identification of the mummy’s sex was made from the CT images. The same holds true for an age estimation.

#### General findings by paleoimaging

**FIGURE 13:**
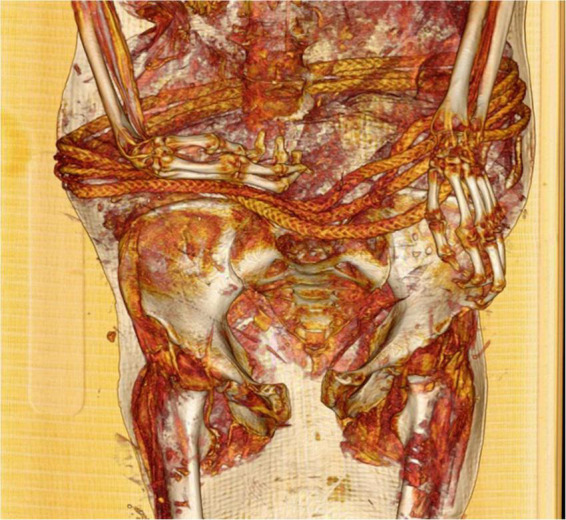
CT-scan (3D-reconstruction) of the female Delémont mummy showing the belt around the waist.

The body, recorded as female, lies in supine position with both arms slightly flexed at the elbow. The right wrist is flexed and the left slightly ulnar deviated. A braided rope is wound around the waist four times, making it a belt as part of the woman’s clothing (Figure 13).

CT-scanning images confirm the records. The body belongs to an adult female as clearly indicated by the typical female external genitalia and the also characteristic form and size of the sciatic notch of the iliac bones. Similarly, the skull provides female features. As to the individual’s age at death, all epiphyses are fused and the cranial sutures appear partly closed indicating an age at death of around 20–30 years. The measurement of the stature indicates that the woman was 161 cm tall (16). The teeth exhibit significant wear and there are some intravital losses (teeth 25, 28, 34, 36, 37, 46, 47) with alveolar recession. However, most of the absent teeth seem to have been lost post-mortem as evidenced by well-formed sockets [teeth 11, 12, 13, 21, 22, 23, 24, 31, 32, 35 (19)]. Furthermore, at the premolar of tooth 25 (left upper jaw) an extensive apical dentogenic abscess is present. As a particular observation, a wooden rod seems to have been inserted postmortem into the spinal canal from C7 to T8, most probably in order to stabilize the spine of a fragile skeleton or mummy. Accordingly, this should have happened long after death—possibly before the shipping of the mummy to Europe. The body has been significantly damaged at some stage. The spine is disrupted at several levels, most prominent at the C7/T1 level with a gap from T1 to T5. The L3 vertebra has been displaced into the abdominal cavity, leaving a gap between L2 and L4. Finally, there is a gap between L5 and the sacrum. The pelvic disruption is noted below.

As to the head and neck region, mummified cerebral tissue is present in the cranial cavity; the eyes are found *in situ* but significantly desiccated. A dislocation of the right temporo-mandibular joint—almost certainly post-mortem—can be appreciated. As stated above, there is disruption of the C7/T1 vertebrae, otherwise there is no abnormality in the cervical region.

**FIGURE 14:**
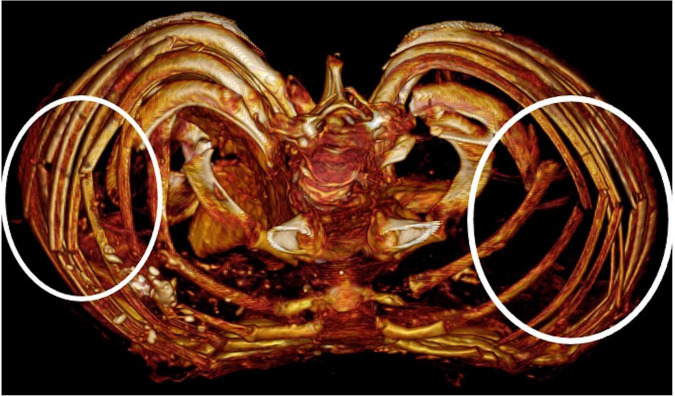
CT scan reconstruction showing bilateral rib fractures without bony reaction (circles).

There is disruption of the upper costo-vertebral joints and fractures/breaks of most ribs laterally without any bone reaction (Figure 14). Naturally mummified organs lie draped over the spine. The abdominal wall is fragmented and within the abdomen a desiccated organ, most likely the liver, is seen. The bony pelvis is disrupted, as is the vertebral column at various levels. As to the internal organs and soft tissues in general, there is no evidence for calcifications, particularly no arteriosclerotic calcium deposits. Several internal organs are present in a significantly shrunken desiccated condition, but without obvious pathological features.

In the upper limbs, there is a fracture of the right clavicle at its lateral end which is without tissue reaction. Although there is no fracture of the left clavicle, there is a fracture of the left scapular blade. The right elbow is dislocated but there are no other skeletal abnormalities in the upper limbs. The right lower limb is intact down to the foot where the os calcis, talus, navicular, and medial cuneiform bones are present, but the rest of the foot is absent. On the left, the upper quarter of the fibula is missing. In the foot, the first ray seems to terminate at the medial cuneiform bone but distal to this, there is a bony structure which does not conform to the shape of the first metatarsal. If anything, the shape is reminiscent of a distal fibula. All toes are absent and the third metatarsal head is missing.

##### Treatment of the mummy

The body is covered in folds of material or some sort of cloak or robe exposing the left shoulder and upper limb and ending distally at the junction of the middle and upper thirds of the tibiae. Within the body cavity is a metal object (a tack or nail) presumably introduced in the relatively recent past. Mummification appears to be natural.

#### Medico-legal evaluation

The Delémont female mummy shows significant post-mortem defects, disruptions, and dislocations of individual bones/skeletal elements, particularly:

– dislocation of the right temporo-mandibular joint with major disruption of the surrounding soft tissues and ligaments;
– disruption of the vertebral column at various levels, particularly at C7/T1
– fracture of the right clavicle without reaction
– fracture of the left scapula without reaction
– dislocation of the right elbow
– disruption of the upper costo-vertebral joints
– numerous fractures of the ribs on both sides laterally, at a site each typical for post mortem compression of the thorax
– disruption of the abdominal wall and the lumbar spine
– disruption and dislocation of the bones of the hip including the symphysis pubis
– significant loss of material at both distal lower extremities

There is no evidence for an intra-vitam (pre-/perimortem) fractures or lesions. All aforementioned lesions are most likely of post-mortem nature; the cause of death remains, therefore, unknown.

## Discussion

### Paleopathology, trauma and human remains

The scientific investigation of mummies provides a particular insight into life, disease, and death of past populations; this holds particularly true for the determination of traumatic injuries and their outcomes. The investigation of trauma residues may provide information about the trauma cause, be it by accident or by human violence, or about the frequency of violence within distinct population groups. A main problem, however, lies in the availability of the most appropriate material: skeletal residues—the most frequent type of tissue remains—excludes soft tissue lesions; mummies may reveal also soft tissue trauma, but complete bodies are much less frequently available than skeletons. In order to overcome the restrictions of tissue accessibility, studies have often been performed on human skulls which are more common in museum collections.

With respect to skeletal material, as a further limitation, bone residues may provide solid data only for those traumatic events that occurred “early” enough ante-mortem to reveal healing/remodeling signs; perimortem lesions must be distinguished from pseudopathology and taphonomic changes (23). The presence of soft tissues may provide additional information on bone lesions—the soft tissue covering broken bones and misalignments visible in radiographs and CT scans may also help identify non-healed osseous trauma. In the distinction between *intra-vitam* and *post-mortem* traumatic lesions, traumatic injuries with a presumed or proven medico-legal background may be of particular interest, as they shed additional light on type and level of interpersonal violence in specific populations (24).

For the elucidation of this context, South American mummies—many of which are excellently preserved—are of particular significance. Since written sources about pre-Hispanic South American populations are limited, any further source of information may be helpful. Usually, South American mummies are of natural, non-artificial origin which reduces post-mortem manipulations of the dead body.

In summary, the precise and circumstantial investigation of each mummy with respect to trauma is of utmost interest. We therefore present here further cases from two distinct museum collections that highlight the scientific potential of transdisciplinary investigation in the analysis of perimortem traumata.

### Evidence for trauma in South American paleopathology

In general, the identification of traumatic injuries has been found in almost all ancient cultures and time periods. The largest database is available for ancient Egyptian mummies and skeletons. While in dry bone traumatic residues can securely be identified as vital only after a survival time of c. 10–20 days (23), in cases with overt adjacent soft tissue traumata, shorter survival periods have been identified (25–27).

The same situation exists for South American mummies (28), with, however, far less data than in Egypt. Likewise, there exist several isolated case reports where a perimortem death due to trauma has been identified (6, 9). Likewise, in a pre-Columbian skull from the Nasca region (ca. 120–750 CE) (9), a rotational trauma of the cervical spine at the atlanto-axial transition zone (C1/C2) with extensive soft tissue bleeding on site and into the skull and with lethal consequences has been diagnosed. Since only the skull was available in this case, no further information is present on an eventual affliction of other body parts or other trauma sequels. In the aforementioned case (6), an almost complete female mummy without provenance (radiocarbon dated to the final pre-Columbian period: 1451–1642 CE), revealed major trauma in the skull; terrace-like fractures of the facial bones, typical for massive hits with a weapon against the skull, were identified. There was no further evidence for any other trauma sequels. The type and the morphology of the lesions were strongly suggestive for a ritual homicide.

As a further single case example, de Souza et al. (29) described a male infantile mummy with an extensive frontal skull defect with adjacent radiating fractures which was covered by overlying intact skin and soft tissues. This clearly suggests a premortal massive strike against the skull in an obviously lethal assault. Another case has been reported by Bianucci et al. (30) who observed a South American mummy head with a soft tissue scar at the right cheek and an underlying fracture of the right maxilla and the mandibular condyle. Accordingly, a pre-mortem blow against the right cheek can be assumed, however, here no cause of death can be indicated.

Beyond these case studies with all their valuable information there exist relatively few series of mummies that have been analyzed to this respect. Herrmann and Meyer (31) investigated 63 mummies (including several mummy fragments), however, “only” by x-ray. Additionally, the analyzed mummy collection came from various locations, populations and time-periods so that the observations do allow only general interpretations. In this series 11 cases provided evidence of pre- or perimortem trauma affecting the skull and/or the postcranial skeleton.

A more recent extensive study of mummies from the site of Ancón, Peru by Watson Jiménez has now been published, providing detailed evidence and CT imaging of mummy bundles from this important funerary site, and includes the analysis of trauma as well as other pathological conditions (32). Several Master’s theses and dissertations, as well as various publications have also appeared in recent years on mummies and skeletal remains from the important site of Puruchuco-Huaquerones, also on the Central Coast of Peru. Many of these focus on trauma and social change in the late Pre-Columbian and early Colonial Period [e.g., (33, 34)].

Recently, Standen et al. (8) describe type and extent of violence in a South American horticulturist population of Northern Chile. On the basis of the human remains of 194 individuals typical evidence for trauma was detected in 40 (21%) of adults, particularly men. Pattern and apparently used weapons suggest interpersonal violence, with 50% (*n* = 20) of trauma appearing fatal. The co-investigation of stable isotope pattern identified local population settings of afflicted persons. These findings suggested that violence occurred between local groups and that social and ecological constraints likely triggered violence within local communities.

### General pathological findings in the three mummies of this study

The three individuals—although obviously coming from various time periods and possibly different locations—are typically naturally mummified human remains with good preservation of soft tissues. All three are adults, two males, one female, were of adult/mature age, with additional non-trauma pathology: all individuals show dental disease with premature loss of teeth and/or age-associated abrasion of enamel; one mummy (“Marburg”) revealed signs of an extensive old-healed pulmonary tuberculosis with focal typical calcifications, extensive intrapulmonary scarring and significant bilateral adhesions of visceral and parietal pleura. There was no evidence for extra-pulmonary tuberculosis. A further individual suffered from calcifying arteriosclerosis (“Délémont male”) typically affecting the aorta and large arteries. The third mummy (“Délémont female”) did not show additional pathology of inner organs.

Beyond these findings, we detected in all three individuals signs of trauma with, however, different evidence as to vitality and period of origin. Two individuals had received vital trauma, while one mummy showed only postmortem alterations.

### Bone “lesions” with clear evidence for postmortem induction (pseudopathology)

These can be identified when osseous “lesions” affect unusual locations (e.g., skull trauma out of the hat brim line in assumed high altitude falling), when irregular osseous discontinuity or dislocation atypical for trauma mechanisms are present or when the bones show a different coloration (20).

**FIGURE 15:**
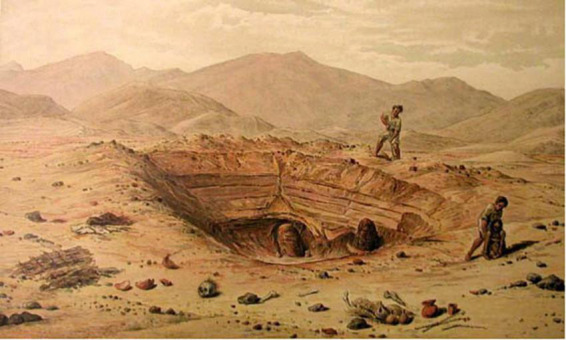
Sketch from the book by Reiss and Stübel (35) on the excavations and findings at the necropolis of Ancon, Peru. As it was typical at the end of the 19th/beginning of the twentieth century, the content of graves was examined on the spot: mummy bundles and objects worth selling were taken away, invaluable items discarded.

Taking these criteria into account, we can attribute potential “lesions” to taphonomic alterations, e.g., during the excavation of the mummies, their transport and/or mishandling during the management. Many such defects seem to have occurred during the excavation—also by grave robberies or intentional opening of burial places. Impressive examples have been e.g., shown by Reiss and Stübel (35) as early as in the late nineteenth century (Figure 15) where disrupted mummy bundles have been shown in a typical burial setting.

### Pre- and perimortem traumata

Both the Marburg mummy and the Delémont male mummy show clear evidence for vital trauma sequels. The Marburg man clearly was a victim of a homicide. The observations on the mummy provide circumstantial evidence that he was stabbed at the right lower back; the deep wound reached the thoracic aorta causing an immediate right hemo-pneumothorax. The laceration of the aorta should have led to rapid hypovolemic shock with almost immediate loss of consciousness. Possibly, the pre-existing old-healed pulmonary tuberculosis may have facilitated a rapid draining of the blood loss out of the corpse, due to a “fixation” of the lung in an expanded form. In consequence, the blood loss may have been much greater than now seen within the thoracic cavity.

Beyond the obviously immediate cause of death, this individual also suffered of repeated skull traumata: a fracture of the right zygoma and a dislocation of the left temporo-mandibular joint. These lesions do not show acute tissue reactions (bone healing signs, massive bleeding residues or extensive disruption of the ligaments/soft tissue structures). Interestingly, the right zygomatic fracture is not associated with any trauma of the right mandible. We, therefore, surmise that the “Marburg man” received a very circumscribed blow to the right zygomatic arch. While the skull lesions are not a direct cause of death, they were most likely part of a complex traumatic event. The same holds true for the dislocation of the left temporo-mandibular joint, where the isolated type of lesion suggests a different traumatic mechanism as on the right side. Hence, we propose that two different *perimortem* attacks against the skull occurred.

With respect to the possible events leading to this individual’s death, we may discuss the following scenarios: First, there may have been one or two assaulters. The fracture of the right upper face indicates an assaulter standing (a) in front of the victim with a backhanded right-handed strike or (b) a left-handed strike on the right side of the victim. The weapon causing this trauma appears to be most likely a club or cudgel leading to blunt trauma. The wound in the right dorsal thorax indicates an assaulter standing behind the victim and stabbing him with a cross strike move from superior right to inferior left, hitting the aorta by the force of the strike. Secondly, two possible scenarios appear likely. (a) One assaulter hit the victim with full force on the head and the second assaulter took the opportunity to stab the victim (who still was standing or kneeing) in the back. (b) The same or another assaulter standing on the right side of the victim struck the head and then turned to the back of the victim and stabbed him or vice versa.

It is likely that the victim was standing or on his knees, because stabbing a victim lying face down would imply a straight dorsal-to-ventral transthoracic stab wound from an assaulter standing above the victim which is less likely, because the assaulter must have used two different weapons shortly after one another (possibly the attacker was ambidextrous).

Besides the aforementioned intra-vitam and post-mortem lesions in the Délemont male individual, there is evidence of sequels of intra-vitam and peri-mortem trauma in this individual. The large skull perforating lesion of the left temporal region and adjacent skull fractures do not show vital reaction and may have occurred peri-mortem. Similarly, the three additional fractures of the left zygomatic arch present with dislocation of a major bone fragment and do not show vital reaction and were most likely inflicted at the time of death.

Besides these recurrent and finally peri-mortem lesions against the skull the mummy reveals a massive trauma against the cervical spine which represents most likely the cause of death. The significant dislocation of the cervical vertebral bodies itself is lethal and may have led to immediate death. The intactness of the perilesional soft tissue strongly argues in favor of an intravital trauma and merely excludes post-mortem defects caused by excavation and/or transportation. It could, however, be possible that the vertebral lesion may have happened intravitally without the pronounced dislocation which may have manifested later, e.g., during preparation of the corps for burial, therefore explaining the lack of major bleeding residues. Also, post-mortem shrinkage of the tissue may have caused pronounced dislocation.

As to the pathogenesis of the defect, a significant flexion trauma may have occurred in an unconscious individual leading to the dislocation of the vertebral bodies. Although this type of lesion is rare, dislocation lesions of the cervical spine in hyperflexion and rotation have been described (36). Further reports additionally indicated the occurrence of similar unusual isolated non-C1/C2 vertebral bone fractures with dorsal translocation (37) or C3-fracture dislocation that even have been survived (38). In summary, we speculate that, while the man was stunned by a blow to the skull, the disruption of the cervical spinal cord occurred by a massive blow. Importantly, the dislocation of the cervical vertebral bodies would have escaped detection in a skeletonized body. Again, the analysis of a well-preserved mummy is essential for a proper diagnosis.

## Conclusion

The three cases described here contribute to the identification and understanding of trauma sequels in South American mummies. While the osseous lesions would also have been identified in skeletonized human remains, both the stabbing and the cervical rotational trauma would have escaped detection in the skeletonized status.

Our observations underline the value of an interdisciplinary approach that takes not only the morphological/radiological features into account, but also the pathophysiologic aspects of trauma mechanisms as they are used in present-day medico-legal case interpretations and evaluations. In summary, only such a combined assay may provide the most precise numbers of trauma sequels available—and may furthermore identify the trauma mechanisms. The analysis of as many cases as possible may indicate the role of trauma in past populations and their interaction in terms of interpersonal violence.

## Data availability statement

The original contributions presented in this study are included in the article/supplementary material, further inquiries can be directed to the corresponding author/s.

## Author contributions

A-MB, RL, OP, JV, RB, IM, MG, and AN: conception of the study, writing first draft, and correction and final draft. A-MB, IM, and MG: historic research. A-MB, OP, and AN: paleopathologic analysis. RL and AN: paleoradiologic study. All authors contributed to the article and approved the submitted version.
